# Recombinant Hendra viruses expressing a reporter gene retain pathogenicity in ferrets

**DOI:** 10.1186/1743-422X-10-95

**Published:** 2013-03-25

**Authors:** Glenn A Marsh, Elena R Virtue, Ina Smith, Shawn Todd, Rachel Arkinstall, Leah Frazer, Paul Monaghan, Greg A Smith, Christopher C Broder, Deborah Middleton, Lin-Fa Wang

**Affiliations:** 1 CSIRO Animal, Food and Health Sciences, Australian Animal Health Laboratory, Geelong, VIC 3220, Australia; 2 Department of Microbiology and Immunology, Uniformed Services University, Bethesda, MD 20814, USA; 3 Program in Emerging Infectious Diseases, Duke-NUS Graduate Medical School, Singapore 169857, Singapore; 4 Current address - Monash Institute of Medical Research, Clayton, VIC 3168, Australia

**Keywords:** Henipavirus, Hendra virus, Reverse genetics, Rescue system, Pathogenesis, Paramyxovirus, Ferret, Zoonotic disease

## Abstract

**Background:**

Hendra virus (HeV) is an Australian bat-borne zoonotic paramyxovirus that repeatedly spills-over to horses causing fatal disease. Human cases have all been associated with close contact with infected horses.

**Methods:**

A full-length antigenome clone of HeV was assembled, a reporter gene (GFP or luciferase) inserted between the P and M genes and transfected to 293T cells to generate infectious reporter gene-encoding recombinant viruses. These viruses were then assessed *in vitro* for expression of the reporter genes. The GFP expressing recombinant HeV was used to challenge ferrets to assess the virulence and tissue distribution by monitoring GFP expression in infected cells.

**Results:**

Three recombinant HeV constructs were successfully cloned and rescued; a wild-type virus, a GFP-expressing virus and a firefly luciferase-expressing virus. *In vitro* characterisation demonstrated expression of the reporter genes, with levels proportional to the initial inoculum levels. Challenge of ferrets with the GFP virus demonstrated maintenance of the fatal phenotype with disease progressing to death consistent with that observed previously with the parental wild-type isolate of HeV. GFP expression could be observed in infected tissues collected from animals at euthanasia.

**Conclusions:**

Here, we report on the first successful rescue of recombinant HeV, including wild-type virus and viruses expressing two different reporter genes encoded as an additional gene cassette inserted between the P and M genes. We further demonstrate that the GFP virus retained the ability to cause fatal disease in a well-characterized ferret model of henipavirus infection despite the genome being an extra 1290 nucleotides in length.

## Background

Hendra virus (HeV) is a zoonotic paramyxovirus harboured by Australian mainland flying foxes from which it is believed to be transmitted directly to horses. HeV first emerged in 1994 in Hendra, a suburb of Brisbane, Australia, leading to an outbreak of acute respiratory disease in 21 thoroughbred horses, of which 14 died [1-3]. During this outbreak, two horse trainers also became infected with the virus, one fatally. In horses, HeV causes a severe, often fatal, febrile illness associated with respiratory and neurological signs [4]. Since its emergence in Queensland, Australia in 1994, HeV spillover from flying foxes to horses has regularly recurred: with an increase in disease events occurring over the past two years [5]. Thirty-nine disease events have occurred resulting in the death or euthanasia of seventy-six horses and one dog, with a case fatality of 75% in the horses.

HeV is the prototype species of the genus *Henipavirus*, within the subfamily *Paramyxovirinae*[6,7]. Nipah virus (NiV) is the only other virus officially classified within the *Henipavirus* genus. NiV was first identified during a major outbreak of acute respiratory disease in pigs in peninsular Malaysia in 1998–99. Over one million pigs were culled in Malaysia to prevent the continued spread of the virus. Over 265 farm and abattoir workers exposed to infected pigs were infected by NiV, resulting in a total of 105 deaths in Malaysia and Singapore [8-10]. Since the outbreak of disease in Malaysia and Singapore, NiV re-emerged in Bangladesh in 2001, with continued re-emergence and human cases almost annually since in Bangladesh and sporadically in India [11,12]. Differences in transmission have also been observed between the Bangladesh and Malaysian strains of NiV. NiV Bangladesh has been shown to cause direct bat-to-human transmission without the involvement of an intermediary or amplifying host. Human-to-human spread of NiV in Bangladesh has also been documented [12-15]. Because of the broad host range and the high mortality rates associated with these viruses, both HeV and NiV have been classified as a biosafety level 4 (BSL-4) agents.

Recently, we described a novel paramyxovirus, Cedar virus (CedPV), which displayed many of the distinguishing characteristics of the henipaviruses, including similar genome length and organisation, it displayed antigenic cross-reactivity with henipaviruses and used the same host cell molecule (ephrin B2) as a receptor for entry during infection [16]. Interestingly in preliminary animal challenge studies, CedPV did not cause disease in ferrets and guinea pigs, both of which are susceptible to fatal disease by the henipaviruses. In addition, a near full length genome sequence has been described for a bat-borne virus from Ghana, Africa [17], that shows around 50% sequence identity with the henipaviruses, including CedPV. Henipa-like viruses have also been detected serologically in bats in Thailand [18], China [19], Madagascar [20] and West Africa [21], with successful virus isolation obtained from Lyle’s flying foxes in Cambodia [22].

Reverse genetics of negative strand RNA viruses allow for the creation of recombinant infectious and replication-competent viruses with specific mutations or insertions. Often, researchers have inserted the green fluorescent protein (GFP) gene into such viruses allowing for the real-time monitoring of virus replication and spread, either within cell culture or *in vivo* within an infected host. The expression of GFP allows for the detection of virus infection in tissues without the need for antibody-specific detection methods. The generation of recombinant henipaviruses will be an extremely powerful tool to monitor viral infections both in real-time for imaging and in a high-throughput approach for screening activities. They will also play a pivotal role in our understanding of pathogenesis of henipaviruses at the molecular level through the generation, rescue and testing of specific mutation variants. Rescue systems have previously been reported for NiV [23-25], here we report the generation of recombinant HeV in which the GFP (HeV-GFP) or firefly luciferase gene (HeV-Luc) has been inserted as an additional transcriptional unit between the P and M genes, and we assess their biological characteristics both *in vitro* and *in vivo*.

## Results and discussion

### Rescue of recombinant viruses

**Figure 1:**
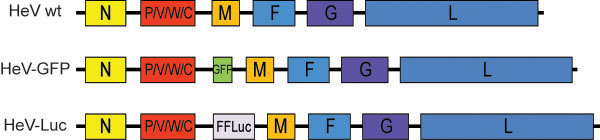
**Schematic representation of the genomes of each of the recombinant viruses.** Full gene cassettes including the 5’UTR from the M gene, the reporter gene and the 3’ UTR of the P gene were inserted for expression of the GFP or luciferase.

A full length antigenome clone of HeV (Hendra virus/horse/1994/Hendra) was prepared and used for modification by the insertion of GFP and firefly luciferase reporter genes. Reporter genes were inserted between the P and M genes, including the untranslated regions from the 3’ end of the P gene and the 5’ end of the M gene (Figure 1). The rule of six was maintained in both the GFP and luciferase constructs (HeV-GFP: 19,524 nucleotides; HeV-Luc: 20,460 nucleotides). The HeV-GFP construct is similar to a NiV-GFP infectious clone previously reported [25], however in the NiV-GFP virus, GFP was inserted between the N and P genes. In this study, the reporter genes were inserted between P and M in order to keep the ratio of N and P consistent with wild-type virus. This was considered important for optimal replication and for maintenance of full virulence. The full HeV-Luc construct at 20,460 nucleotides resulted in the largest reported genome of a paramyxovirus.

Rescue of recombinant HeV for wild-type, HeV-GFP and HeV-Luc viruses were achieved by co-transfection of human 293T cell in 12.5 cm^2^ flasks with the full length antigenome plasmid, N, P and L protein expression vector and pCAGGS T7 RNA polymerase. Two days after transfection, Vero cells (5 × 10^5^ cells) were added to the 293T cell monolayers. In successful experiments, CPE was obvious within 3 days following the addition of the Vero cells, with CPE reaching maximal levels around 7 days post transfection. Supernatants were collected from the transfected cell monolayers and clarified by centrifugation and used to infect Vero cell monolayers to prepare virus stocks for further study.

### *In vitro* characterisation

**Figure 2:**
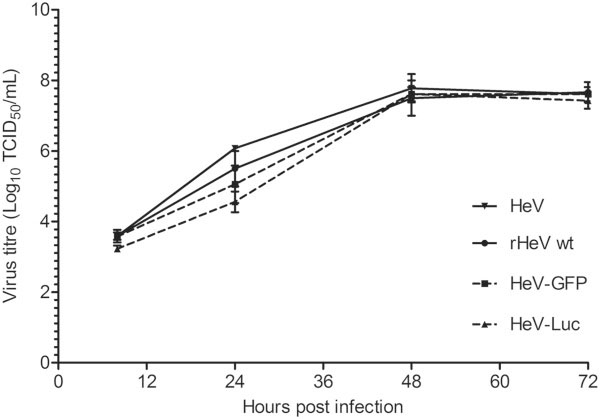
**Kinetics of recombinant virus growth in Vero cells.** Vero cells were infected at an MOI of 0.01 and samples collected for titration at 8, 24, 48 and 72 hours post infection. Results are expressed as the average of 3 independent infections, with error bars representing standard deviation.

Growth characteristics of the 3 recombinant viruses were compared in Vero cells (Figure 2). Replication of each virus followed a similar pattern, at 24 hours, the recombinant viruses show slightly lower titres compared to the parental virus however, and all reached a maximal titre of 3–5 × 10^7^ TCID_50_/mL by 48 hours post infection.

**Figure 3:**
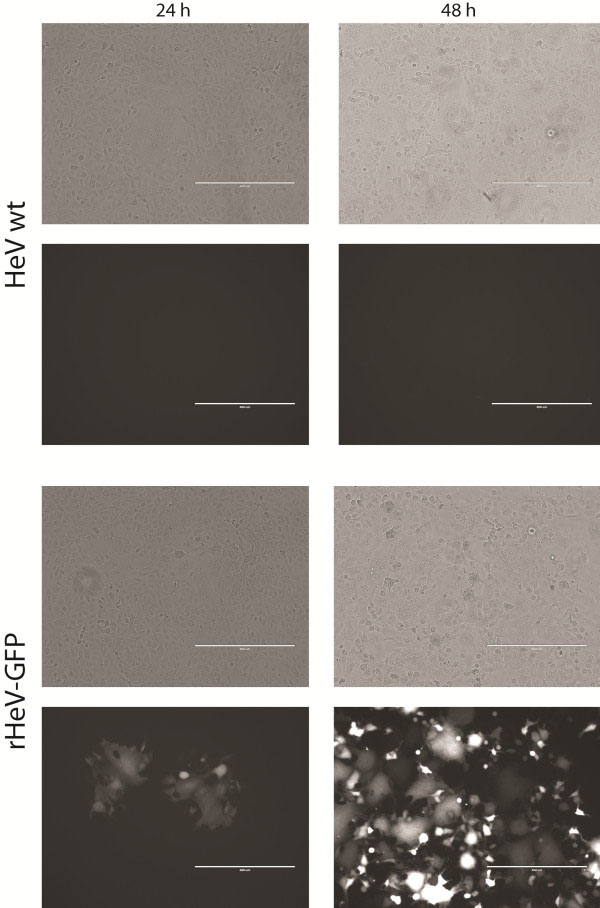
**Expression of GFP in Vero cells infected with HeV-GFP recombinant virus.** Vero cells were infected at an MOI of 0.05 and imaged using a AMG EVOS FL fluorescent inverted microscope with a monochrome camera at 24 and 48 hr post infection.

In order to determine expression of the GFP reporter genes within infectious virus, cells were infected with HeV-GFP an MOI of 0.01 and imaged by fluorescent microscopy at 24 and 48 hours post infection. GFP positive cells were visible at 24 hours post infection and by 48 hours all cells were fluorescent, consistent with the spread of virus and infection of all cells by 48 hours (Figure 3). Stable GFP expression by HeV-GFP was assessed by serially passing the virus 5 times to fresh cell. Strong GFP expression was maintained through the 5 passes. The GFP expression allows for real-time monitoring of virus replication and spread in infected cells.

**Figure 4:**
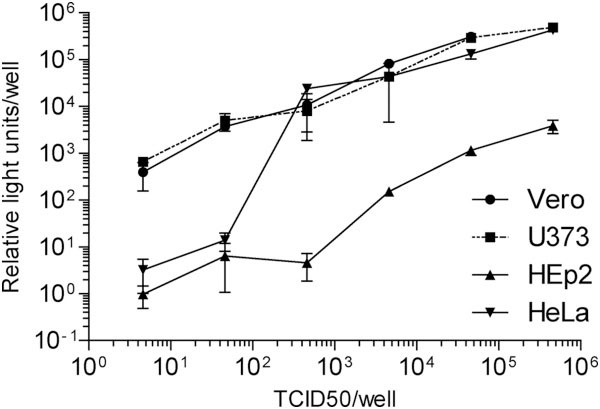
**Expression of luciferase by the recombinant HeV-Luc.** Vero, HeLa, HEp2 and U373 human cells were infected with various amounts of HeV-Luc. At 24 hours post infection, luciferase activity in cells was measured by lysing cells using BrightGlo reagent (Promega) and reading on a Synergy H4 microplate reader (Biotek Instruments Inc). Results represent the average of 4 independent infections with error bars representing standard deviation. All values have been normalised to uninfected cells (relative light units of 1).

Expression of luciferase by the recombinant HeV-Luc was assessed by infecting cells with various amounts of virus for 24 hours. Luciferase activity in cells was then measured by lysing the cells using BrightGlo reagent (Promega) and reading on a Synergy H4 microplate reader (Biotek Instruments Inc). As virus dose increased, the level of luciferase increased proportionately (Figure 4). Differences were observed when different cell lines were used in this study, demonstrating the usefulness of this virus to quantitatively assess the infection and/or replication efficiency of HeV in different cell lines. Luciferase expression can be used for high-throughput monitoring of virus replication, hence they are a useful tool for screening drug candidates. This virus is currently being used for a genome-wide RNAi screen of human genes critical for HeV replication.

### *In vivo* characterisation

**Figure 5:**
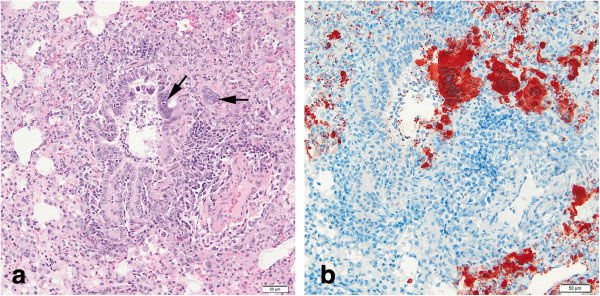
**Necrotising bronchoalveolitis with epithelial syncytia in lung of ferret infected with HeV-GFP virus.** (**a**) H and E and (**b**) immunohistochemical staining of HeV N protein showing presence of antigen in red. Arrow indicates syncytia.

**Figure 6:**
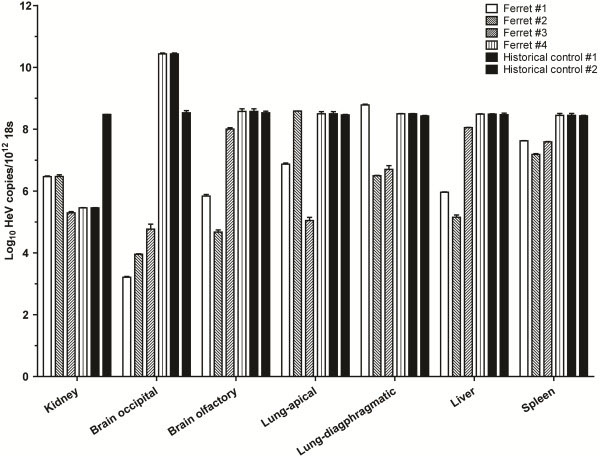
**Relative abundance of HeV-GFP RNA in different tissues of the ferret.** Various tissues were collected at post mortem examination and analysed for HeV-GFP viral load by real-time PCR. Values are expressed relative to ribosomal 18S copies.

To determine whether the insertion of a reporter gene into HeV attenuated the virus *in vivo*, ferrets were exposed to 5,000 TCID_50_ of HeV-GFP virus by the oronasal route. This dose was previously determined to be lethal for ferrets using the parental wild-type isolate of HeV: two ferrets became febrile on day 6 post challenge and were euthanased on reaching pre-determined humane endpoints on day 6 or day 7 post challenge [26]. Lesions in these animals included systemic vasculitis, bronchoalveolitis, glomerular necrosis, and lymphoid necrosis, associated with syncytial cell formation and HeV antigen deposition in lesional tissues. Disease progression with the HeV-GFP virus was consistent with that observed using wild-type virus, with ferrets becoming febrile from 3 to 6 days post challenge and reaching pre-determined humane endpoints on day 7 or 8 post challenge (2 ferrets on each day). Gross post mortem and microscopic pathology findings were also similar to the previous report of HeV infection in ferrets. These include hemorrhagic lymph nodes and petechial hemorrhages throughout the lungs and renal cortices, with necrotising rhinitis, systemic vasculitis, syncytial cell formation, necrotising bronchoalveolitis (Figure 5), lymphadenitis, and splenic and renal glomerular necrosis accompanied by HeV antigen deposition in affected tissues. Real-time PCR analysis of HeV RNA in clinical samples and in tissues collected at euthanasia matched that observed previously (Figure 6) [26].

**Figure 7:**
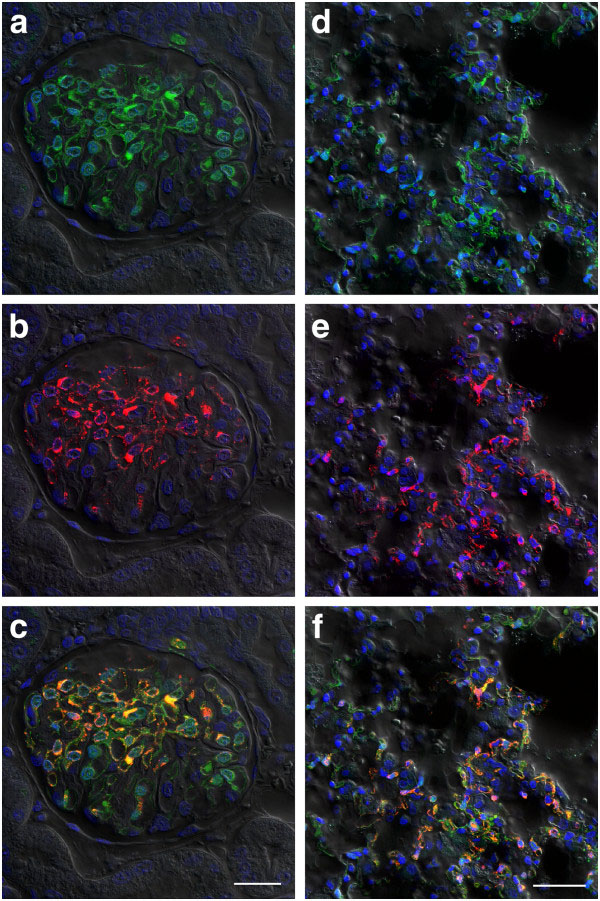
**Expression of GFP in tissues sectioned from HeV-GFP challenged ferrets.** Ferrets were challenged with 5,000 TCID_50_ of HeV-GFP and succumbed to disease on days 7 and 8. At necropsy, kidney (**a**,**b**,**c**) and lung (**d**,**e**,**f**) samples were collected, fixed with paraformaldehye, sectioned and imaged by confocal microscopy. To assess sensitive of GFP expression (green; a and d) to traditional staining techniques, HeV nucleocapsid protein (red; b and e) was co-stained using rabbit anti- HeV N sera detected with anti-rabbit conjugated Alexafluor 568. Composite images (c and f) show both GFP and HeV N labelling. Scale bars: c =25 μm, f = 40 μm.

Imaging of tissue sections from lung, kidney and spleen of HeV-GFP infected ferrets by confocal microscopy showed significant numbers of GFP-containing cells. To determine whether all infected cells were GFP-positive, sections were co-stained for HeV nucleocapsid (Figure 7). This showed that imaging of GFP expression in tissues for HeV infection was as sensitive as immunofluorescent labelling of tissue sections.

## Conclusions

Here we report the first successful rescue of replication-competent recombinant HeV, including both wild-type HeV as well as recombinant viruses encoding either the GFP or luciferase genes by genetic insertion between the P and M genes. The rescue of the HeV-Luc virus resulted in a paramyxovirus with the largest functional reported genome to date. Challenge of ferrets with HeV-GFP demonstrated that the insertion of GFP into the virus genome did not moderate its virulence, with GFP expression being observed in tissues collected from ferrets at euthanasia. The HeV-Luc virus is currently being utilized as a powerful tool for studying host genes important for the replication of HeV (unpublished data).

## Methods

### Cells and virus

293T cells (ATCC CRL-11268) were maintained in Dulbecco’s Modified Eagle’s Medium (DMEM) high glucose (Invitrogen), supplemented with 10% fetal bovine serum (FBS), 100 U/mL penicillin, 100 mg/mL streptomycin, 4 mM L-glutamine. African green monkey (Vero – ATCC CCL-81) cells were maintained in EMEM supplemented with 10% FBS, 100 U/mL penicillin, and 100 mg/mL streptomycin. HeV (Hendra virus/horse/1994/Hendra isolate, GenBank Accession AF017149) was isolated in Vero cells from the lung of a horse infected in the Brisbane outbreak in 1994 and was passaged and triple plaque purified in Vero cells. All work with live HeV was carried out within the BSL4 facilities at the Australian Animal Health Laboratory, Geelong Australia.

### Generation of plasmids and rescue of recombinant HeVs

Vero cells were infected with HeV and when maximal CPE was observed cell supernatants were collected and virus concentrated by ultracentrifugation (280,000 *g* for 45 minutes). Pellet was then resuspended in buffer RLT (Qiagen) and removed from the BSL4 laboratory. RNA was extracted using a Qiagen RNEasy kit and reverse transcribed to cDNA. Using a panel of PCR primers, large (3–5 kb) fragments were generated by PCR and cloned into pOLTV5 [27] between a T7 RNA promoter and a hepatitis delta virus ribozyme. Full open reading frames for HeV N, P and L genes, the essential components needed for viral RNA transcription and replication, were cloned into pTM-I, a T7 driven mammalian expression plasmid. In addition, the T7 RNA polymerase gene was PCR amplified from BSR/T7 cells and cloned into pCAGGS. All plasmid inserts were sequenced to obtain at least 2-fold sequence coverage to ensure no mutations relative to the reference sequences.

To generate recombinant viruses, 1 × 10^6^ 293T cells were transfected with pTM-I HeV N (1.25 μg), pTM-I HeV P (0.8 μg), pTM-I HeV L (0.4 μg), full-length HeV genome plasmid (3.5 μg) and pCAGGS T7 (2 μg) using 7 μL Lipofectamine 2000 (Invitrogen). Media was changed on cells at 6 hours post transfection and then 5 × 10^5^ Vero cells were added to transfected 293T cells after 2 days. Cells were observed for 7 days for the generation of syncytia, at which time, supernatant was passaged to fresh Vero cells (75 cm^2^ flasks) to amplify recombinant virus. When maximal CPE was obtained, supernatants were clarified by low speed centrifugation (1000 *g*), aliquoted, stored at −80°C and the 50% tissue culture infective dose (TCID_50_)/mL calculated using the Reed and Muench method [28].

### Animal studies

Procedures involving live animals were approved by the Commonwealth Scientific and Industrial Research Organisation, Australian Animal Health Laboratory and Animal Ethics Committee. Four male ferrets, aged 12–18 months, were exposed oronasally to 5000 TCID_50_ of HeV-GFP. This challenge dose was selected as the wild-type isolate has been shown to be lethal in ferrets at this dose (unpublished observations). Ferrets were monitored daily for indications of disease including fever and alertness. Euthanasia was performed at predetermined humane endpoints as previously described [29]. Shedding samples, blood and urine were collected on days 3 and 6 post-exposure as well as immediately prior to euthanasia and various tissues collected at post mortem examination.

### Imaging of tissues

Samples of brain, lung, spleen and kidney tissues from HeV-GFP infected ferrets were dissected into approximately 1 cm cubes and fixed for 48 hr in 4% paraformaldehyde in phosphate buffered saline (PBS). They were stored at 4°C in PBS. Sections were cut at 70 μm on a Leica VT1000 vibrating microtome (Leica Microsystems). Sections were blocked in PBS containing 0.5% bovine serum albumin (PBS/BSA) overnight and then incubated in rabbit anti-HeV N protein diluted 1:1000 in PBS/BSA. Following 3 × 10 min washes in PBS, the bound antibody was detected with goat anti-rabbit IgG conjugated to Alexa 568 (Life Technologies) diluted 1:200 in PBS/BSA, followed by 3 × 10 min washes in PBS. Nuclei were labelled by incubation of sections in a 1:1000 dilution of DAPI (Sigma, Sydney) for 30 min and following 2 × H_2_O washes, the sections were mounted in Vectashield (Vector laboratories). Coverslips were sealed with nail varnish and the sections imaged with a Leica SP5 confocal microscope.

## Competing interests

The authors declare that they have no competing interests.

## Authors’ contributions

GAM conceived the study, carried out the molecular work, *in vitro* characterisation, processed animal tissues, analysed samples and drafted the manuscript. ERV participated in the molecular work. IS provided clones and participated in the molecular work. ST participated in the molecular work. RA carried out the animal infection trials. LF assisted with the animal work. PM performed the confocal imaging work. GAS provided clones and participated in the molecular work. CCB conceived the study, provided funds and edited the manuscript. DM designed the animal study, provided veterinary pathological assistance and drafted the manuscript. LFW conceived the study and drafted the manuscript. All authors read and approved the final manuscript.
